# Uptake of moss‐derived human recombinant GAA in *Gaa* ^−/−^ mice

**DOI:** 10.1002/jmd2.12203

**Published:** 2021-02-01

**Authors:** Stefan Hintze, Paulina Dabrowska‐Schlepp, Birgit Berg, Alexandra Graupner, Andreas Busch, Andreas Schaaf, Benedikt Schoser, Peter Meinke

**Affiliations:** ^1^ Friedrich‐Baur‐Institute at the Department of Neurology University Hospital, Ludwig‐Maximilians‐University Munich Munich Germany; ^2^ eleva GmbH Freiburg Germany

**Keywords:** enzyme replacement therapy, glycogen storage disease type II, moss‐GAA, Pompe disease

## Abstract

Pompe disease, an autosomal recessive lysosomal storage disorder, is caused by deficiency of lysosomal acid alpha‐glucosidase (GAA). On cellular level, there is lysosomal‐bound and free accumulation of glycogen and subsequent damage of organelles and organs. The most severe affected tissues are skeletal muscles and heart. The only available treatment to date is an enzyme replacement therapy (ERT) with alglucosidase alfa, a recombinant human GAA (rhGAA) modified with mannose‐6‐phosphate (M6P), which is internalized via M6P‐mediated endocytosis. There is an unmet need to improve this type of therapy, especially in regard to skeletal muscle. Using different tissue culture models, we recently provided evidence that a moss‐derived nonphosphorylated rhGAA (moss‐GAA), carrying a glycosylation with terminal *N*‐acetylglucosamine residues (GnGn), might have the potential to improve targeting of skeletal muscle. Now, we present a pilot treatment of *Gaa* ^−/−^ mice with moss‐GAA. We investigated general effects as well as the uptake into different organs following short‐term treatment. Our results do confirm that moss‐GAA reaches the target disease organs and thus might have the potential to be an alternative or complementary ERT to the existing one.

## 1 INTRODUCTION

Pompe disease (also glycogen storage disease type II; OMIM #232300) is a lysosomal storage disease, which can be clinically distinguished by a spectrum ranging from a severe infantile onset form (IOPD) to a later‐onset form (LOPD).^1^, ^2^ The main symptoms of IOPD patients are cardiomyopathy and muscular hypotonia with cardiorespiratory failure usually causing death in untreated patients within the first year of life.^3^, ^4^ In LOPD patients, the disease manifests as a multisystemic disorder with a predominant involvement of skeletal muscles. Particularly respiratory muscle weakness causes a premature death in LOPD patients.^5^, ^6^

Pompe disease is caused by recessive mutations in the *GAA* gene, resulting in deficiency of the encoded lysosomal hydrolase acid alpha‐glucosidase (GAA).^7^, ^8^ To date there is only one approved treatment available: enzyme replacement therapy (ERT) with alglucosidase alfa, a recombinant human GAA (rhGAA).^9^ Outcomes of this treatment have been shown to vary among patients, and it appears that in most cases the effect is not a reversal but an attenuation of the disease progression, especially considering skeletal muscle.^10^, ^11^ Therefore, there is an ongoing unmet medical need to develop enhanced, alternative therapies.

It is thought that one of the reasons for the limited efficacy of the current ERT is the relatively low abundance of the cation‐independent mannose‐6‐phosphate receptor (CI‐MPR) on the skeletal muscle surface.^12^, ^13^ CI‐MPR is the cellular receptor mediating the cellular uptake of alglucosidase alfa, which is carrying mannose 6‐phosphate (M6P) glycans.^14^

Using tissue culture system, we recently showed that nonphosphorylated rhGAA carrying terminal *N*‐acetylglucosamine residues (GnGn), produced using a moss‐based expression system (moss‐GAA), was successfully taken up into undifferentiated myoblasts as well as differentiated myotubes. Moreover, in direct comparison to alglucosidase alfa, we observed an improved uptake of moss‐GAA into myotubes.^15^ These results indicated that alternative glycosylation pattern might provide an improved targeting of skeletal muscle in Pompe disease.^15^ To further test whether the cellular uptake of moss‐GAA was not biased by environmental conditions of muscle tissue culture, we performed an initial, narrow organ distribution study investigating delivery of moss‐GAA to selected tissues of *Gaa* ^−/−^ mice.

## 2 MATERIALS AND METHODS

### 2.1 Animals/mouse model

The *Gaa* ^−/−^ mice used^16^ were obtained from The Jackson Laboratory (https://www.jax.org/; stock no. 004154). For the uptake experiment, 3 months old mice received a total of 3 weekly tail vein i.v. bolus injections of either vehicle (25 mM Na‐phosphate‐buffer pH 6.2) or moss‐GAA. Animals were sacrificed 24 hours after the last injection. Age matches wild‐type (WT) animals of the same strain background were used as controls.

All animal experiments were performed in accordance with the national (Tierschutzgesetz and Versuchstierverordnung) and European regulations (European Union directive for the Protection of Animals Used for Scientific Purposes (2010/63/EU) and were approved by the local authorities (Regierung von Oberbayern, reference number ROB‐55.2‐2532.Vet_02‐18‐178).

### 2.2 Production and purification of Moss‐GAA

The cultivation of the moss strain producing moss‐GAA with terminal N‐acetylglucosamine residues (GnGn) was done as described in the literature.^17^ Purification was performed as described in the literature,^15^ moss‐GAA was concentrated to approx. 14 mg/mL for use in in vivo study and stored at −80°C until use.

### 2.3 Tissue processing

For analyses with the GAA activity assay, glycogen assay and western blot, tissues were snap frozen in liquid nitrogen and stored at −80°C. For further processing, tissues were grinded to powder using a porcelain mortar and pestle in liquid nitrogen. Fifty milligram of tissue powder were dissolved in 250 μL of distilled water, ultra sonicated (Sonopuls ultrasonic homogeniser HD2070 with sonotrode MS73, Bandelin), and centrifuged for 10 minutes at 4°C and 10  000 g. Protein concentration of the resulting supernatant was determined using a Qubit 3.0 Fluorometer and Quant‐iT Qubit Proteinassay‐Kit (Q33212).

Tissues used for periodic acid–schiff (PAS) and HE staining were embedded in OCT, snap frozen in liquid nitrogen, and stored at −80°C. Tissue sections were cut to 10 μm tissue sections using cryostat (HM505E; Microm, Walldorf, Germany) at −26°C and mounted on glass slides (Double frosted microscope slides; Fisher Scientific).

### 2.4 GAA activity assay

For GAA activity measurement, 20 μL of tissue extract (4 μg/μL) and 80 μL of reaction buffer (0.25 mM 4‐methylumbelliferyl alpha‐d‐glucopyranoside, 56 mM citric acid, 88 mM Na2HPO4, 0.4% BSA) were mixed (10 seconds at 900 rpm) and incubated for 1 hour at 37°C. After the reaction was stopped (stop buffer: 0.1 M Glycin, 0.1 M NaOH), GAA activity was measured using a Tecan Infinite M200Pro plate reader (orbital mixing step for 5 seconds, amplitude 1.5 mm, excitation: 360 nm, emission: 450 nm, gain: 50). All samples have been measured three times in technical triplicates.

### 2.5 Glycogen assay

Tissue extracts were diluted 1:20 in distilled water, boiled for 10 minutes, and centrifuged at 4°C and 13  000 g. Of the resulting supernatant, 40 μL were used either directly or diluted for incubation with 10 μL amyloglucosidase (freshly prepared 800/U mL in sodium acetate buffer pH 5.0) or 10 μL 0.5 M sodium acetate buffer (pH 5.0). Samples were incubated for 1 hour at 50°C followed by 15 minutes of denaturation (100°C) and 1 hour at 4°C. To this samples, 200 μL Glucose Assay Reagent (Sigma) were added followed by 15 minutes incubation at room temperature. To measure the absorbance at 340 nm a Tecan Infinite M200Pro plate reader was used. Glycogen content for each sample was calculated based on a standard curve and protein concentration used for each sample. All samples have been measured three times in technical triplicates.

### 2.6 PAS staining

Sections were first hydrolyzed for 5 minutes in 1% periodic acid solution and then washed for 5 minutes in 35°C running tap water. Direct after an additional washing step in distilled water, the slides were placed in Schiff's reagent for 15 minutes. Followed by washing steps in running tap water and distilled water, the slides were placed for 1 minute in Mayer's haematoxylin solution to counterstain for the nuclei. Prior to dehydration in 2x 70% EtOH, 2x 80% EtOH, and 5 minutes in 96% EtOH, the slides were rinsed in warm tap water for 10 minutes. Finally, slides were stored in xylol before they were covered with Eukitt and a cover slide.

### 2.7 Western Blot

Proteins from whole protein extracts were separated by SDS gel electrophoresis using self‐prepared gels (10%). Proteins were transferred via wet‐blot on nitrocellulose membranes (0.45 mm pore size). Membranes were blocked with using 5% skim milk in 1x TBS/0.1% Tween 20. Following primary antibodies were used: mouse anti GAA (Abnova, H00002548‐B01P), and rabbit anti GAPDH (Cell Signalling, D16H11) were used. As secondary antibodies we used donkey anti‐mouse IRDye 800CW, donkey anti‐rabbit IRDye 680RD. All western blot images were obtained using a Licor FC. Quantification was done using the Licor ImageStudio Software.

### 2.8 Microscopy and image analysis

Images were obtained using an Olympus IX83 inverted microscope equipped with a 0.95 NA 40x objective and a digital camera (UC90, Olympus).

### 2.9 Statistical analysis

To determine statistical significance, we applied the two‐tailed Student's *t*‐test. *P*‐values below or close to .05 are displayed in the figures.

## 3 RESULTS

### 3.1 Study plan and general observations

**FIGURE 1:**
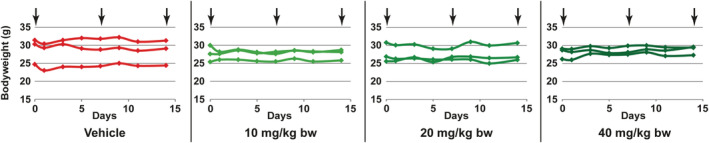
Weight development of mice during treatment. Arrows indicate time points of injection

For treatment, we separated *Gaa* ^−/−^ mice into four groups each containing three individuals. The respective groups were administered either with buffer used as dissolvent for moss‐GAA (vehicle) or with differing amounts of moss‐GAA per bodyweight (10 mg/kg bw; 20 mg/kg bw; 40 mg/kg bw). Additionally, two untreated wild type (WT) mice were used for analyses as a complementary control. Originally, four, weekly injections were planned for treated groups. However, while after first two injections (days 0 and 7), no adverse reaction of mice was observed, following the third injection (day 14), all animals administered with moss‐GAA were dazed for about 2 hours and one animal of each dose group died within 1 hour. After about 2 hours behaviour of the surviving mice was normal again. This observation indicates an immunogenic reaction to a repeated application of a foreign protein. The bodyweight of all mice remained stable for the duration of treatment (Figure 1). Haematoxylin and eosin staining of spleen, liver, muscle, and heart of all animals (the ones that died after the third injection as well as the ones that survived it) was performed. We did not find any pathological changes (data not shown).

### 3.2 Organ distribution

Considering the observed immunogenic reaction, all surviving mice were sacrificed within 24 hours after the third injection and selected tissues: quadriceps, triceps, heart, liver, and spleen were analysed.

**FIGURE 2:**
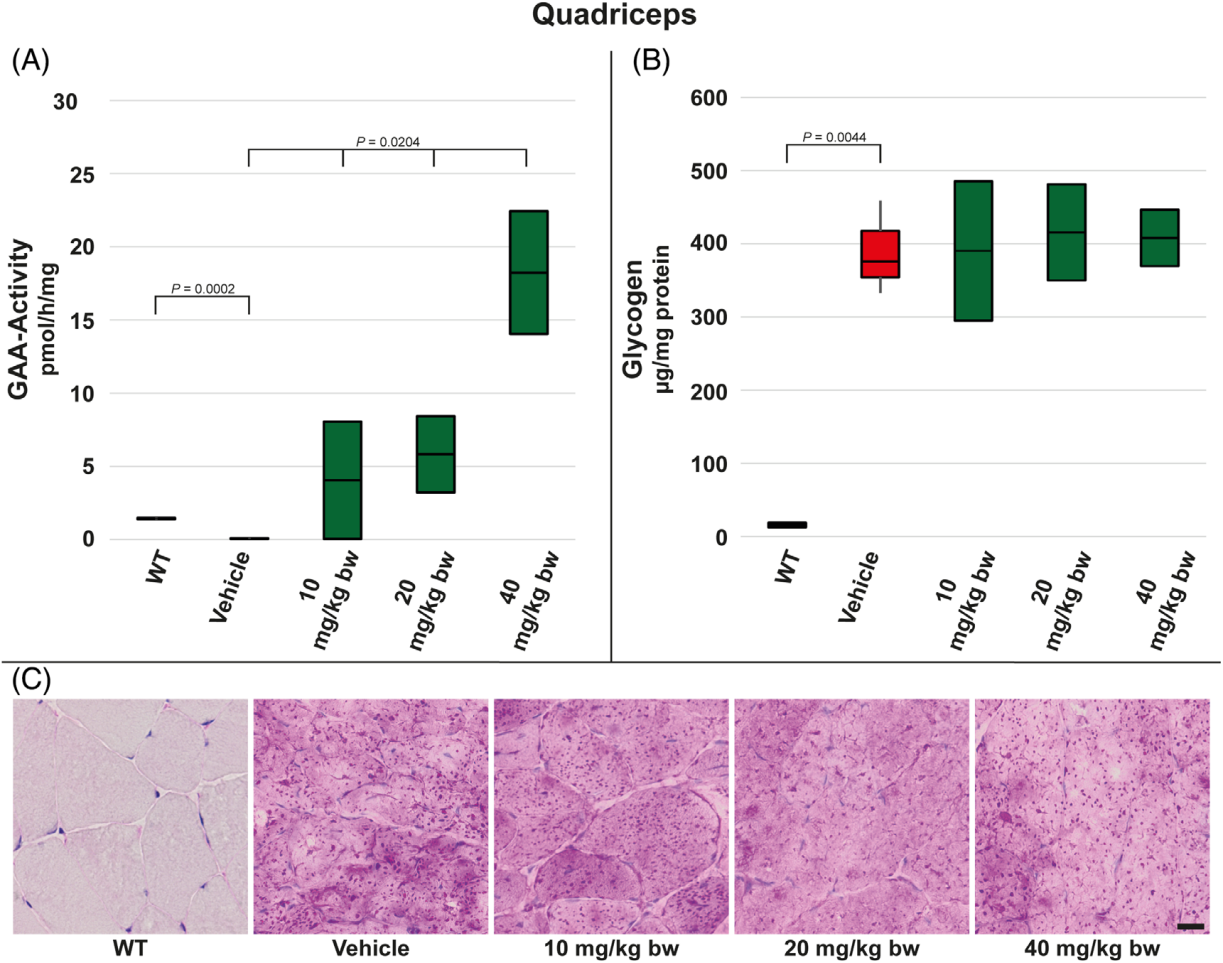
Analysis of quadriceps muscle for WT mice and *Gaa* ^−/−^ mice treated with buffer only (vehicle) and increasing amounts of moss‐GAA: A, GAA‐activity; B, Glycogen content; and C, PAS staining. Scale bar: 20 μm

In quadriceps muscle GAA activity was significantly reduced in vehicle‐treated *Gaa* ^−/−^ mice compared to WT animals. In all groups of moss‐GAA‐treated mice, the measured GAA activity in quadriceps tissue was elevated in a dose‐dependent manner with the GAA activity in groups 20 mg/kg bw and 40 mg/kg bw being significantly higher than in WT animals (approx. 4‐fold and 13‐fold, respectively) (Figure 2A). Additionally measured glycogen content in vehicle‐treated *Gaa* ^−/−^ mice was as expected significantly increased compared to WT animals. Three injections of moss‐GAA did not lead to measurable reduction of glycogen concentration irrespective of dose (Figure 2B). This was confirmed by PAS‐staining, which showed the presence of glycogen accumulation in untreated as well as moss‐GAA‐treated quadriceps muscle of all *Gaa* ^−/−^ mice but not in WT animals (Figure 2C).

**FIGURE 3:**
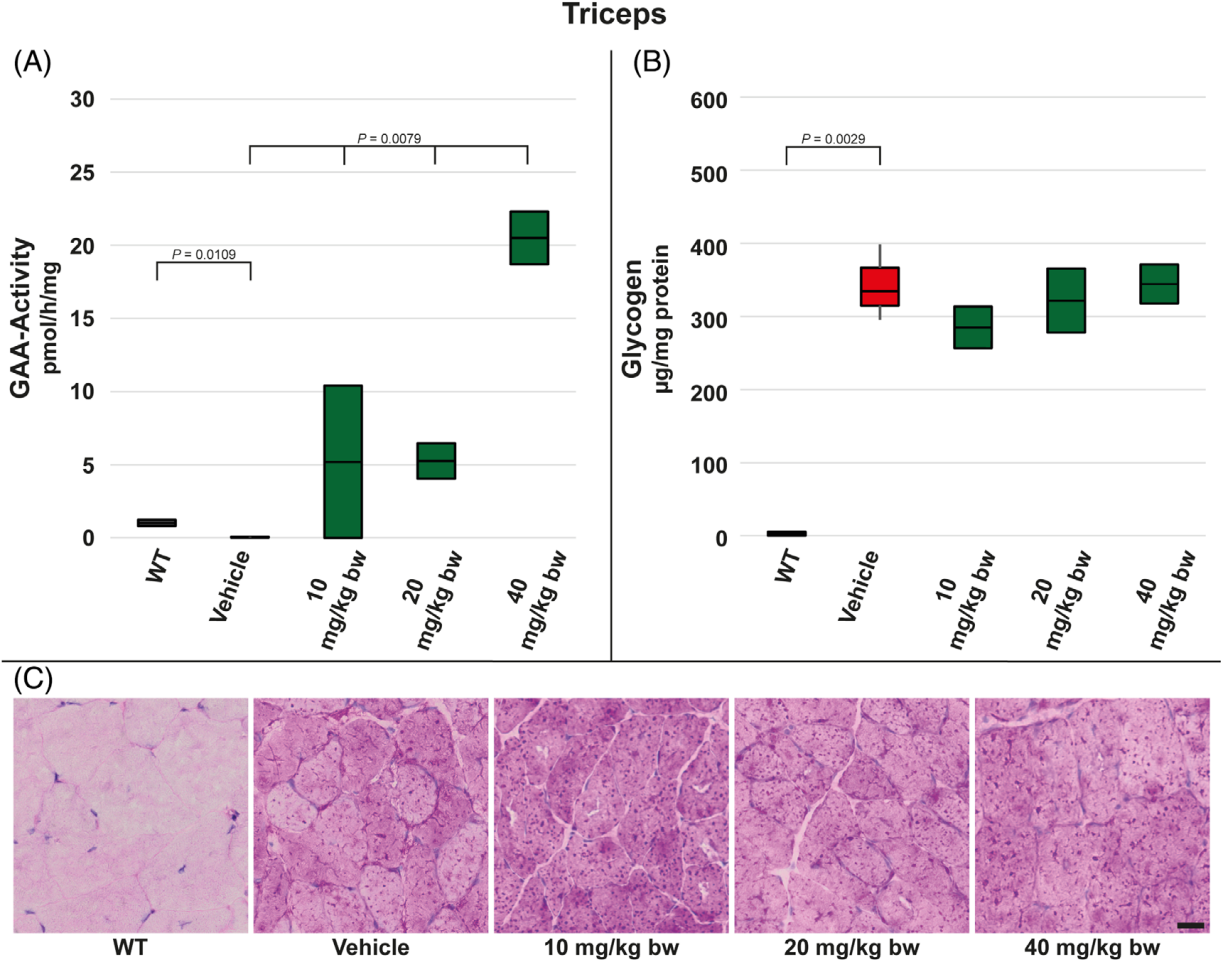
Analysis of triceps muscle for WT mice and *Gaa* ^−/−^ mice treated with buffer only (vehicle) and increasing amounts of moss‐GAA: A, GAA‐activity; B, Glycogen content; and C, PAS staining. Scale bar: 20 μm

In triceps muscle measured GAA activity reflected results from quadriceps muscle: significantly reduced in vehicle‐treated *Gaa* ^−/−^ mice compared to WT animals and elevated in all moss‐GAA‐treated groups in a dose‐dependent manner. A significant elevation was achieved in mice treated with 20 mg/kg bw (approx. 5‐fold vs WT) and 40 mg/kg bw (approx. 18‐fold vs WT) moss‐GAA (Figure 3A). Similarly like in case of quadriceps muscle, treatment with moss‐GAA did not lead to the reduction of glycogen content in triceps tissue (Figure 3B,C).

**FIGURE 4:**
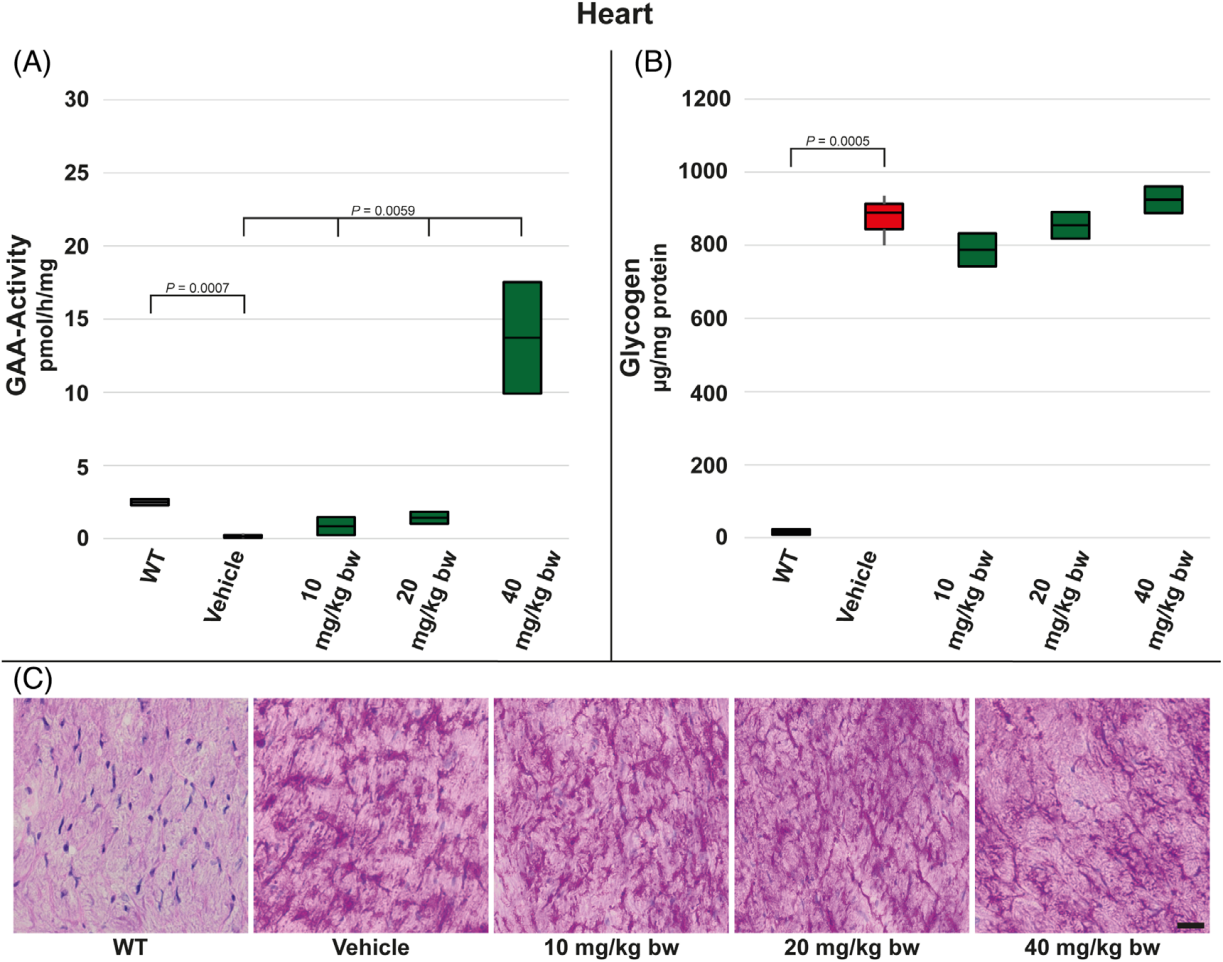
Analysis of heart muscle for WT mice and *Gaa* ^−/−^ mice treated with buffer only (vehicle) and increasing amounts of moss‐GAA: A, GAA‐activity; B, Glycogen content; and C, PAS staining. Scale bar: 20 μm

Next to skeletal muscles, heart tissue was analysed. Comparing vehicle‐treated *Gaa* ^−/−^ mice to WT controls, measured GAA activity was significantly reduced. Moss‐GAA treatment increased the enzyme activity in all dose groups; however, significant elevation vs WT‐level (approx. 6‐fold) was achieved only in the highest dose group with 40 mg/kg bw (Figure 4A). Similarly to the tested skeletal muscles, glycogen levels were elevated in vehicle‐treated *Gaa* ^−/−^ mice compared to WT and did not change following moss‐GAA treatment (Figure 4B C).

**FIGURE 5:**
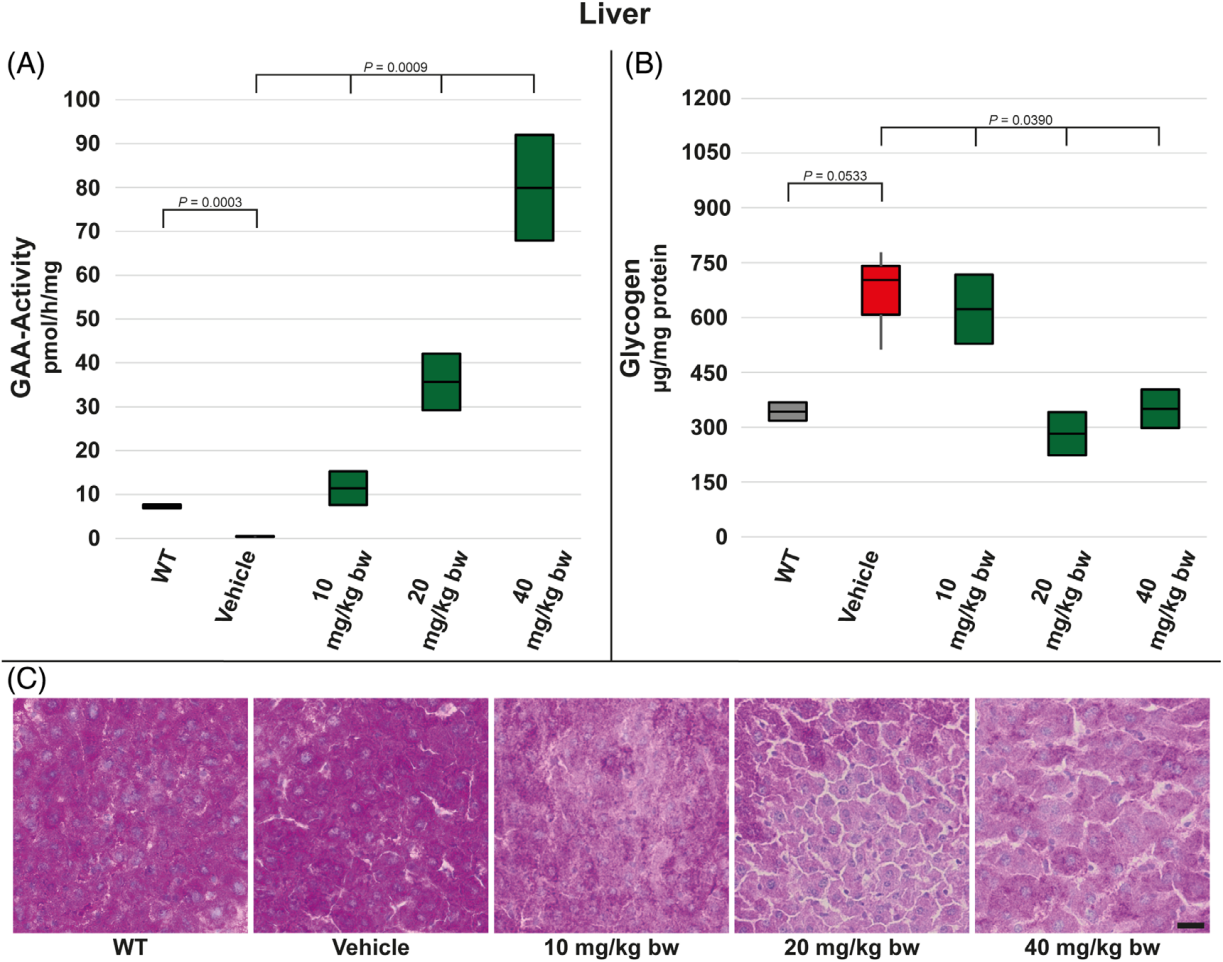
Analysis of liver for WT mice and *Gaa* ^−/−^ mice treated with buffer only (vehicle) and increasing amounts of moss‐GAA: A, GAA‐activity; B, Glycogen content; and C, PAS staining. Scale bar: 20 μm

Apart from muscle tissues, we analysed liver and spleen. In case of liver, GAA activity was significantly reduced in vehicle‐treated *Gaa* ^−/−^ mice compared to WT animals. Like it was the case with investigated muscle tissues, moss‐GAA treatment did also lead to increase in GAA activity in liver, with significant change vs WT groups in all tested dose groups—10, 20 and 40 mg/kg bw (approx. 1.5‐, 5‐, and 10‐fold, respectively) (Figure 5A). In contrast to analysed muscle tissues, glycogen levels in liver of *Gaa* ^−/−^ mice treated with 20 mg/kg and 40 mg/kg moss‐GAA did drop to WT levels following three injections (Figure 5B). We could confirm this result by PAS staining which showed less glycogen accumulation in all dose groups (Figure 5C).

**FIGURE 6:**
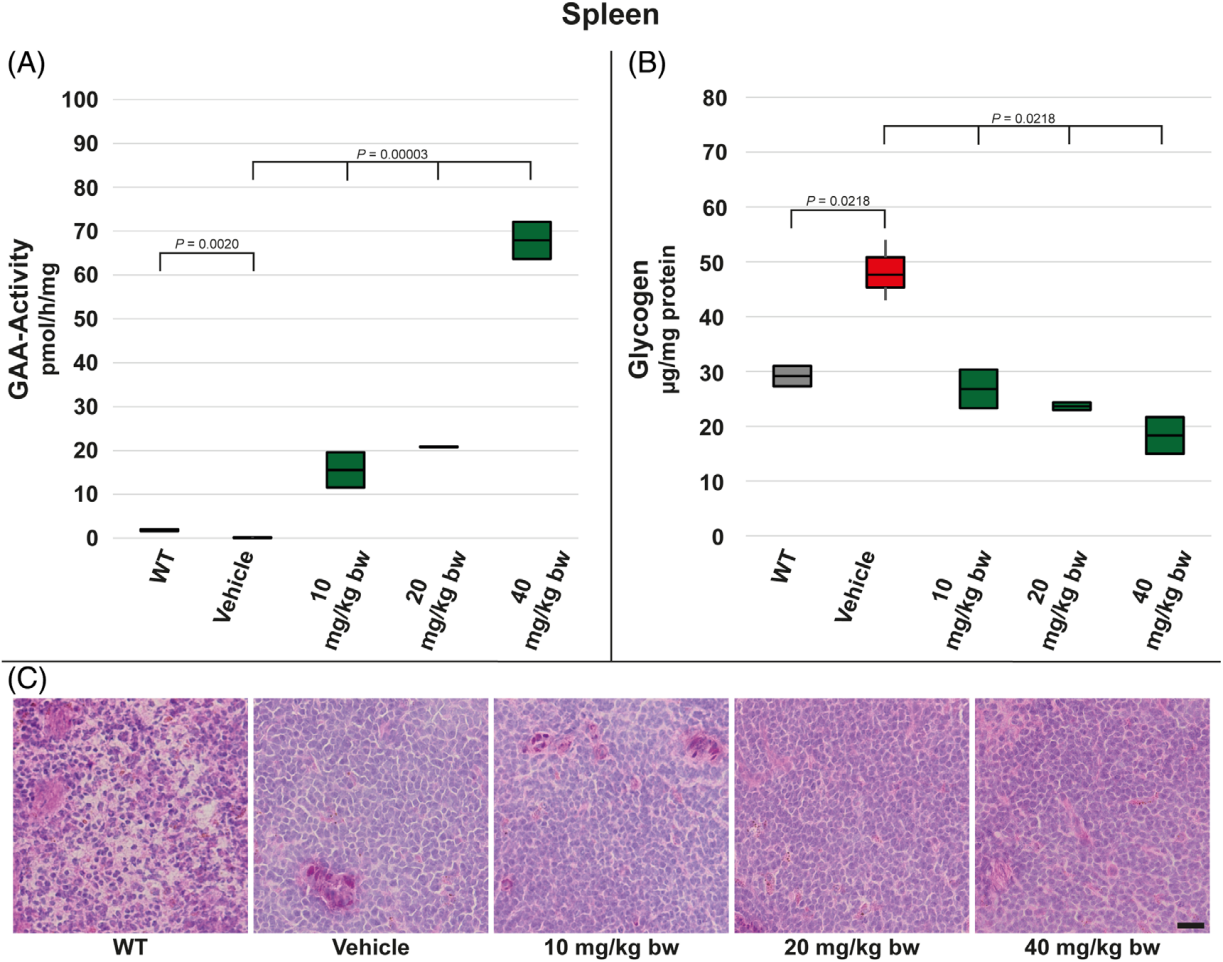
Analysis of spleen for WT mice and *Gaa* ^−/−^ mice treated with buffer only (vehicle) and increasing amounts of moss‐GAA: A, GAA‐activity; B, Glycogen content; and C, PAS staining. Scale bar: 20 μm

In case of spleen, as with other analyzed organs, we could observe a decreased GAA‐activity in vehicle‐treated *Gaa* ^−/−^ mice compared to WT controls. Similarly to liver moss‐GAA administration led to significantly higher enzyme activity than in WT animals in all three dose groups (Figure 6A). Measured glycogen levels were low, when compared to other organs, but the elevation in vehicle‐treated *Gaa* ^−/−^ mice compared to WT animals was obvious. Treatment with all three moss‐GAA doses resulted in a restoration of WT glycogen level (Figure 6B). Due to generally low primary tissue levels of glycogen, PAS staining was not informative in spleen (Figure 6C).

**FIGURE 7:**
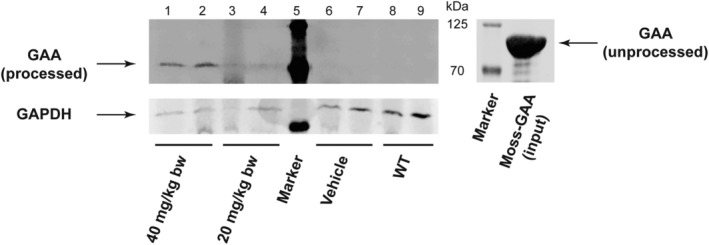
GAA processing: western blot analysis of quadriceps muscle for *Gaa* ^−/−^ mice treated with buffer only (vehicle) and different amounts of moss‐GAA. The moss‐derived GAA precursor used for treatment is loaded as control (0.7 μg). For each tissue lysate, 30 μg of total protein were loaded

In addition to the measurement of GAA activity, which provides an indirect read‐out of GAA uptake, we wanted to know whether the 110 kDa pro‐protein form of moss‐GAA is being correctly processed to the mature lysosomal form of GAA (~76 kDa) post organ uptake. For this, we tested lysates from quadriceps muscle (vehicle‐treated *Gaa* ^−/−^ mice, 20 mg/kg bw, and 40 mg/kg bw moss‐GAA‐treated *Gaa* ^−/−^ mice) using GAA western blotting. In the corresponding tissue from animals treated with 40 mg/kg moss‐GAA, we could clearly identify a band with a size fitting mature GAA (~76 kDa, lanes 7 and 8) while the majority of the moss‐derived GAA used for treatment has the size of the protein precursor (~110 kDa, lane 4) (Figure 7).

## 4 DISCUSSION

The aim of our study was to investigate the uptake and indications for efficacy of moss‐GAA in selected organs of *Gaa* ^−/−^ Pompe mice. We recently showed that moss‐GAA, which is a moss‐produced, nonphosphorylated rhGAA carrying terminal *N*‐acetylglucosamine residues, has the potential to provide an improved targeting of skeletal muscle in a tissue culture model in comparison to the current state of care with alglucosidase alfa.^15^ Considering this to be a pilot “proof‐of‐principle” experiment, we decided to use a small number of animals (three animals per group) and three different dosages.

To elaborate whether moss‐GAA triggers any immune response in a similar manner as alglucosidase alfa^18^ in *Gaa* ^−/−^ mice, we decided not to use any additional immunosuppressant treatment for this pilot experiment. In hindsight, this turned out to be a crippling factor for the assessment of efficacy potential of moss‐GAA in this study. While all moss‐GAA‐treated animals tolerated well the first two injections, the third administration caused a strong immunogenic reaction, leading to death of one individual in each group. In the same time, the vehicle‐treated animals did not show any adverse reaction. The fact that all moss‐GAA‐treated animals were dazed following the third injection supports the notion of an anaphylactic shock type of reaction due to the presence of the GAA protein representing an unknown and additionally species foreign protein structure to the knockout animals. Thus, we conclude that moss‐GAA is similar to alglucosidase alfa in this respect and for future experiments, an additional immunosuppressant (eg, diphenhydramine) has to be considered. Data from animals, which died post third injection, were not included in analysis for organ distribution of moss‐GAA as we could not exclude a GAA contamination from blood remaining in small blood vessels—different modified rhGAA have been shown to remain in blood for up to 20 hours or even longer.^19^

To investigate the uptake of moss‐GAA in quadriceps, triceps, heart, liver, and spleen, we used a GAA activity assay. In all tissues, we observed a dose‐dependent increase in GAA activity upon treatment with moss‐GAA. In skeletal muscles, a higher variation of GAA activity in the lowest dose group was determined. This could be due to the very limited injection volume (we decided to use a constant concentration of moss‐GAA for all injections and varied the injected volume) and possible variability in the actual injected amount of moss‐GAA. For all tissues with the exception of heart tissue, we reached with all tested doses higher GAA activities than in WT controls. In heart tissue only the highest dose (40 mg/kg) exceeded WT GAA activity levels. This shows not only that moss‐GAA is efficiently taken up into various tissues but also that phosphorylation does not seem to be essential for the uptake of GAA enzyme into disease relevant tissues. Additionally, different glycosylation pattern of moss‐GAA compared to alglucosidase alfa results in a differing tissue targeting. While the direct comparison to alglucosidase alfa in our study is missing, it is known that this rhGAA is especially effective in targeting of the heart, whereas skeletal muscle effectively addressed. The reason for this is thought to be the relatively low abundance of the CI‐MPR receptor on the skeletal muscle surface.^12^, ^13^ Moss‐GAA on the other hand seems to be more effectively targeting skeletal muscles than heart as we do significantly exceed WT GAA activity levels starting already with the 20 mg/kg bw dose (the lowest values in these groups are resulting in more than a doubling of GAA activity level vs WT), whereas in heart a definite increase was observed only with the highest dose of 40 mg/kg bw.

Apart from the GAA activity, we also investigated changes in glycogen levels post moss‐GAA administration. In heart tissue as well as skeletal muscles, we saw no changes in glycogen levels after 14 days of treatment, however in liver and spleen glycogen amounts dropped to WT levels. This obvious discrepancy between the increased GAA activity in the organs and simultaneous lack of efficacy in glycogen reduction could be explained by several factors. First of all, the immune response following the third injection likely resulted in a reduced delivery of freely available moss‐GAA due to antibody binding in blood. Second of all, the actual time of treatment, which had to be restricted to three injections (3 weeks) was probably too short to see effects on glycogen levels in tissues with long regeneration time, as heart and skeletal muscle.

The precursor of mature lysosomal GAA is a 110 kDa protein containing *N*‐linked carbohydrates modified with mannose 6‐phosphate groups, which is in turn processed to a 76 kDa mature protein.^14^ This processing mechanism increases the affinity of GAA for glycogen and thus its ability for glycogen degradation.^14^ The modification of the GAA precursor with mannose‐6‐phospate has been assumed to be necessary for lysosomal targeting, although a phosphomannosyl receptor‐independent transport could not be excluded.^14^ We could confirm that moss‐GAA is successfully processed from the 110 kDa precursor to the 76 kDa mature protein in quadriceps, thus we conclude that there exists a mannose‐6‐phosphate independent mechanism for GAA targeting, which is affecting not only its uptake but also its processing.

In summary, we could show that moss‐GAA, however missing phosphorylated glycans, is efficiently taken up in all investigated tissues and achieves overall higher activity increase vs WT in skeletal muscles then in heart tissue. Moreover, correct intracellular processing of moss‐GAA could be demonstrated. A broader and longer in vivo study with application of adequate immunosuppressants would be necessary to properly assess the efficacy potential of moss‐GAA in comparison to alglucosidase alfa. Nevertheless, already accumulated results indicate that using a nonphosphorylated rhGAA with terminal *N*‐acetylglucosamine residues (GnGn) could provide an additional or complementary ERT to the current standard of care.

## CONFLICT OF INTEREST

Paulina Dabrowska‐Schlepp, Birgit Berg, Andreas Busch and Andreas Schaaf are employees of eleva GmbH. This work was supported by eleva GmbH. SH, AG, PM, BS report no conflicts of interest.

## AUTHOR CONTRIBUTIONS

Alexandra Graupner and Stefan Hintze performed the experiments. Stefan Hintze, Peter Meinke, and Benedikt Schoser were involved in conception and design of the experiments, in analysis and interpretation of the data, and contributed to the writing of the manuscript. Paulina Dabrowska‐Schlepp, Birgit Berg, Andreas Busch, and Andreas Schaaf were involved in conception and design of the experiments, provided moss‐GAA material for the study and contributed to the writing of the manuscript. This work was supported by an unrestricted research grant to Benedikt Schoser by eleva GmbH.

## ANIMAL CONSENT

All animal experiments were performed in accordance with the national (Tierschutzgesetz and Versuchstierverordnung) and European regulations (European Union directive for the Protection of Animals Used for Scientific Purposes (2010/63/EU) and were approved by the local authorities (Regierung von Oberbayern, reference number ROB‐55.2‐2532.Vet_02‐18‐178).
